# Prediction of 5-year postoperative survival and analysis of key prognostic factors in stage III colorectal cancer patients using novel machine learning algorithms

**DOI:** 10.3389/fonc.2025.1604386

**Published:** 2025-07-14

**Authors:** Wei Zhang, Yan Li, Jinghan Jia, Yuhang Yang, Yuyuan Hu, Yanhong Wang, Jinxi Wang

**Affiliations:** ^1^ Division of Colorectal Surgery, Third Hospital of Shanxi Medical University, Shanxi Bethune Hospital, Shanxi Academy of Medical Sciences Tongji Shanxi Hospital, Taiyuan, China; ^2^ Neurology, Third Hospital of Shanxi Medical University, Shanxi Bethune Hospital, Shanxi Academy of Medical Sciences Tongji Shanxi Hospital, Taiyuan, China

**Keywords:** colorectal cancer, machine learning, SEER database, prognostic model, survival prognosis

## Abstract

**Objective:**

This study explores the predictive value of clinical and socio-demographic characteristics for postoperative survival in stage III colorectal cancer (CRC) patients and develops a 5-year postoperative survival prediction model using machine learning algorithms.

**Methods:**

Data from 13,855 stage III CRC patients who underwent surgery were extracted from the SEER database. Key variables, including marital status, gender, tumor location, histological type, T stage, chemotherapy status, age, tumor size, lymph node ratio, and others, were collected. Data were split into a 7:3 training-validation ratio. Optimal cutoff points for age, tumor diameter, and lymph node ratio were determined using X-tile software. Independent prognostic factors for postoperative survival in stage III colorectal cancer patients were identified through univariate and multivariate logistic regression as well as Lasso regression analyses. These factors were incorporated into machine learning models, including logistic regression, decision tree, LightGBM, and others. ROC curves, calibration curves, and decision curve analysis were used to assess model performance. External validation was performed using data from Shanxi Bethune Hospital.

**Results:**

Optimal cutoff points were identified for age (65, 80 years), tumor size (29 mm, 74 mm), and lymph node ratio (0.11, 0.49). Both multivariate logistic regression and Lasso regression consistently identified marital status, tumor location, histological type, T stage, chemotherapy, radiotherapy, age, maximum tumor diameter, lymph node ratio, serum carcinoembryonic antigen (CEA) level, perineural invasion, and tumor differentiation as independent prognostic factors for 5-year postoperative survival in patients with stage III colorectal cancer (P < 0.05). The models showed excellent predictive performance with AUC values ranging from 0.766 to 0.791 in the validation cohort. Age, lymph node ratio, chemotherapy, and T stage were key factors. External validation confirmed model accuracy and clinical applicability.

**Conclusion:**

This study developed and validated an interpretable machine learning model that predicts the 5-year postoperative survival of stage III CRC patients, offering potential for personalized treatment plans.

## 1 Introduction

Colorectal cancer (CRC) is one of the most prevalent malignant tumors globally (1), with both incidence and mortality rates steadily rising, particularly in developing countries. Research suggests that factors such as lifestyle changes, dietary patterns, and population aging collectively contribute to the continued increase in CRC incidence (2). In China, the number of CRC cases and deaths has reached 555,000 and 286,000, respectively, ranking second in cancer incidence and fifth in cancer mortality (3).

Stage III CRC is a locally advanced malignancy characterized by tumor invasion through the bowel wall and regional lymph node involvement, without distant metastasis. Surgical treatment combined with adjuvant chemotherapy is the standard approach for this stage; however, significant variability in survival outcomes persists among patients (4). The current prognostic evaluation based on the American Joint Committee on Cancer (AJCC) TNM staging system (5) has notable limitations. Although this system has undergone several revisions, it primarily focuses on anatomical tumor characteristics (such as the extent of metastasis, lymph node involvement, and the depth of primary tumor invasion), while overlooking key prognostic factors such as age, tumor size, and adjuvant therapy. Consequently, its predictive capability remains limited (6, 7).

As a significant branch of computer science, machine learning (ML) represents an advanced technology for pattern recognition and knowledge acquisition, built on the foundation of extensive datasets (8, 9). By leveraging vast data resources, ML techniques employ feature extraction and pattern analysis to identify crucial variables and uncover hidden relationships within the data, providing a robust scientific basis for decision-making. Previous studies have shown that machine learning algorithms outperform traditional methods in complex disease detection systems, such as CRC, by utilizing collective decision-making mechanisms that mitigate the limitations of single-model generalization abilities (10). Several studies have explored the application of machine learning in CRC screening and prognosis evaluation, confirming the feasibility of this approach. For instance, ensemble learning methods, including logistic regression, decision trees, and random forests, have demonstrated promising results in tumor staging and postoperative recurrence prediction (11, 12). These methods integrate clinical, imaging, and pathological data to uncover underlying patterns and provide personalized treatment recommendations for patients.

This study utilizes data extracted from the Surveillance, Epidemiology, and End Results (SEER) database (13) for stage III CRC patients post-surgery, and constructs a 5-year survival prediction model using ensemble machine learning algorithms. Additionally, a comprehensive analysis of the key prognostic factors influencing postoperative survival is conducted.

## 2 Materials and methods

### 2.1 Patient cohorts

#### 2.1.1 SEER cohort

This study is based on SEER*Stat (version 8.4.4) and accesses data from the SEER cancer database (http://SEER.cancer.gov/). Specifically, data from the SEER Research Data, 17 Registries, Nov 2023 Sub (2000-2021) version were used for statistical analysis. The study focuses on colorectal cancer (CRC) cases diagnosed between 2010 and 2015. The primary site codes “C18.0, C18.2-19.7, C19.9, C20.9” were selected from the Primary Site-Labeled field, and cases with surgical codes “10-80” from the RX Summ—Surg Prim Site field were included. Data were standardized and filtered according to the 7th edition of the AJCC TNM staging system. Inclusion criteria: (1) Pathologically confirmed CRC diagnosis; (2) Patients with stage III CRC; (3) CRC as the only primary malignancy, excluding interference from other types of cancer; (4) Patients who survived or died from CRC. Exclusion criteria: (1) Missing clinical information (e.g., grade, tumor size, histology); (2) Lack of follow-up data or missing survival time or follow-up period information.

#### 2.1.2 External cohort

A retrospective analysis was performed on data from 185 stage III CRC patients who underwent surgical treatment at the General Surgery Department of Shanxi Bethune Hospital from January 2017 to December 2019. The inclusion and exclusion criteria were consistent with those used for the SEER cohort. Data from the external cohort were utilized for independent external validation.

### 2.2 Data classification

In the SEER database, 14 variables were selected for analysis, including marital status, gender, tumor location, pathological type, T stage, radiotherapy status, chemotherapy status, age, maximum tumor diameter, number of lymph nodes examined, number of positive lymph nodes, serum CEA levels, perineural invasion (PNI) status, and tumor differentiation. The specific definitions are as follows: (1) Marital status: At the time of CRC diagnosis, categories such as “single or living with partner,” “divorced,” “separated,” and “widowed” were all categorized as “unmarried”; (2) Tumor location: C18.0, C18.2, C18.3 represent the right colon; C18.4 represents the transverse colon; C18.5, C18.6 represent the left colon; C18.7 to C20.8 represent the rectosigmoid junction; C20.9 represents the rectum; (3) Pathological type: 8140–8389 correspond to adenocarcinoma, and other codes represent non-adenocarcinoma; (4) Radiotherapy and chemotherapy status: Due to the limitations of the SEER database, specific details of anticancer treatment regimens are not available, and the order of chemotherapy, radiotherapy, and surgery is not clearly recorded; (5) Lymph node ratio (LNR): The ratio of the number of positive lymph nodes to the total number of lymph nodes examined; (6) Serum CEA levels: Based on the highest CEA level recorded by the medical institution before treatment, >5 ng/ml is considered “positive,” and ≤5 ng/ml is “negative”; (7) PNI: Based on histopathological evidence, the presence of nerve invasion was assessed and categorized as “yes,” “no,” or “unknown”; (8) Tumor differentiation: Based on the maturity of tumor cells, classified as “Grade I (well-differentiated),” “Grade II (moderately differentiated),” “Grade III (poorly differentiated),” “Grade IV (undifferentiated),” and “unknown.” All stage III CRC postoperative patients included in the study were divided into a training set (70%) and a validation set (30%). The optimal cutoff points for patient age, maximum tumor diameter, and LNR were calculated using the X-tile software (version 3.6.1) on the training set (14). X-tile is a bioinformatics tool based on the minimum *p*-value principle, which identifies optimal cutoff points in continuous variables that are significantly associated with prognosis through Kaplan-Meier analysis. The software offers advantages such as intuitive visualization, no need for predefined thresholds, and ease of use, and it has been widely applied in cancer prognosis studies (14). However, a major limitation of X-tile is the potential risk of overfitting, particularly when applied to small sample sizes or in the absence of external validation, which may compromise the generalizability of the identified cutoffs. Therefore, in this study, we performed additional modeling using multiple algorithms and validated the results with external data to ensure the robustness of the conclusions.

### 2.3 Study outcomes

This study uses colorectal cancer-specific survival (CSS) as the primary endpoint, defined as the time from diagnosis to death due to CRC or the date of the last follow-up. Survival status was categorized into two groups: “alive” and “death due to CRC,” for statistical analysis. The SEER database provides direct data on overall survival. For patients at Shanxi Bethune Hospital, survival follow-up was conducted according to AJCC guidelines, with regular follow-ups performed through the hospital’s electronic medical system and by telephone. Follow-up data were censored as of December 31, 2024.

### 2.4 Data preprocessing

During the data preprocessing stage, we first assessed the missingness of all variables. For variables with a missing rate of less than 5%, we chose to directly remove the incomplete records without imputation. For variables with a missing rate of 5% or higher, we applied mean or mode imputation based on the type of variable—mean imputation for continuous variables and mode imputation for categorical variables—in order to retain more samples and minimize information loss.

### 2.5 Model establishment

This study employs univariate and multivariate logistic regression analyses to select feature variables from the training set, constructing predictive models using nine machine learning algorithms: Logistic Regression (LR), Decision Tree (DT), Elastic Net (Enet), K-Nearest Neighbors (KNN), Light Gradient Boosting Machine (LightGBM), Random Forest (RF), eXtreme Gradient Boosting (XGboost), Support Vector Machine (SVM), and Multilayer Perceptron (MLP). Model performance is evaluated using metrics including accuracy, precision, recall, F1 score, and the area under the receiver operating characteristic curve (AUC). The AUC value ranges from 0.0 to 1.0, with a value closer to 1.0 indicating better model discrimination, while a value near 0.5 suggests that the model’s predictive ability is similar to random guessing. Based on the AUC value and other evaluation metrics from the validation set, the optimal predictive model is selected. Furthermore, calibration and decision curve analyses (DCA) are performed to assess the model’s calibration and clinical utility. Additionally, Shapley Additive Explanations (SHAP) are employed for model interpretability analysis. To ensure the model’s robustness and clinical applicability, an external validation cohort of stage III CRC postoperative patients from Shanxi Bethune Hospital is used, with corresponding ROC (Receiver Operating Characteristic) curves, calibration curves, and DCA curves generated to comprehensively evaluate model performance.

### 2.6 Statistical methods

Data collection for this study was performed using SEER*Stat software (version 8.4.4), and basic statistical analysis was conducted using R software (version 4.3.3). Qualitative data were described using frequency statistics and proportions (%), while intergroup differences were tested using the chi-square test (χ²). During the data analysis, univariate logistic regression was initially employed to select variables, followed by multivariate logistic regression to include the selected variables in the model. The odds ratio (OR) and its corresponding 95% confidence interval (95% CI) were calculated, with statistical significance set at P < 0.05. To further validate the robustness of variable selection, Lasso regression was also applied for comparative analysis. By introducing regularization, Lasso effectively selects relevant variables, reduces model complexity, and minimizes the risk of overfitting. Additionally, multicollinearity diagnostics were performed by assessing the correlation between variables and calculating the variance inflation factor (VIF). A nomogram was constructed using the “rms” package based on the results of the multivariate analysis, and a more flexible and intuitive dynamic nomogram was generated using the “DynNom” package. For non-tree-based machine learning models that are sensitive to feature scaling, such as KNN, SVM, logistic regression, and MLP, all input variables were standardized using Z-score normalization prior to model training to ensure numerical stability and comparability of results. In this study, logistic regression, DT, ENet, KNN, LightGBM, RF, XGboost, SVM, and MLP machine learning algorithms, along with the ROC curve, calibration curve, DCA for external validation, and SHAP model interpretability, were implemented using the “tidymodels” package. To evaluate the impact of different clinical characteristics on the 5-year survival time of patients, Kaplan-Meier survival analysis was performed. Cancer-specific survival time (defined as the time from diagnosis to death due to colorectal cancer or the date of last follow-up) was used as the time variable, and survival status (alive/deceased) was used as the outcome variable. Patients were grouped according to key variables such as LNR, T stage, age group, and chemotherapy status. Survival curves were plotted, and the log-rank test was used to compare survival differences between groups. All statistical tests were two-sided, and a P-value < 0.05 was considered statistically significant.

## 3 Results

### 3.1 Demographic and clinical characteristics

#### 3.1.1 SEER cohort patient data

**Figure 1:**
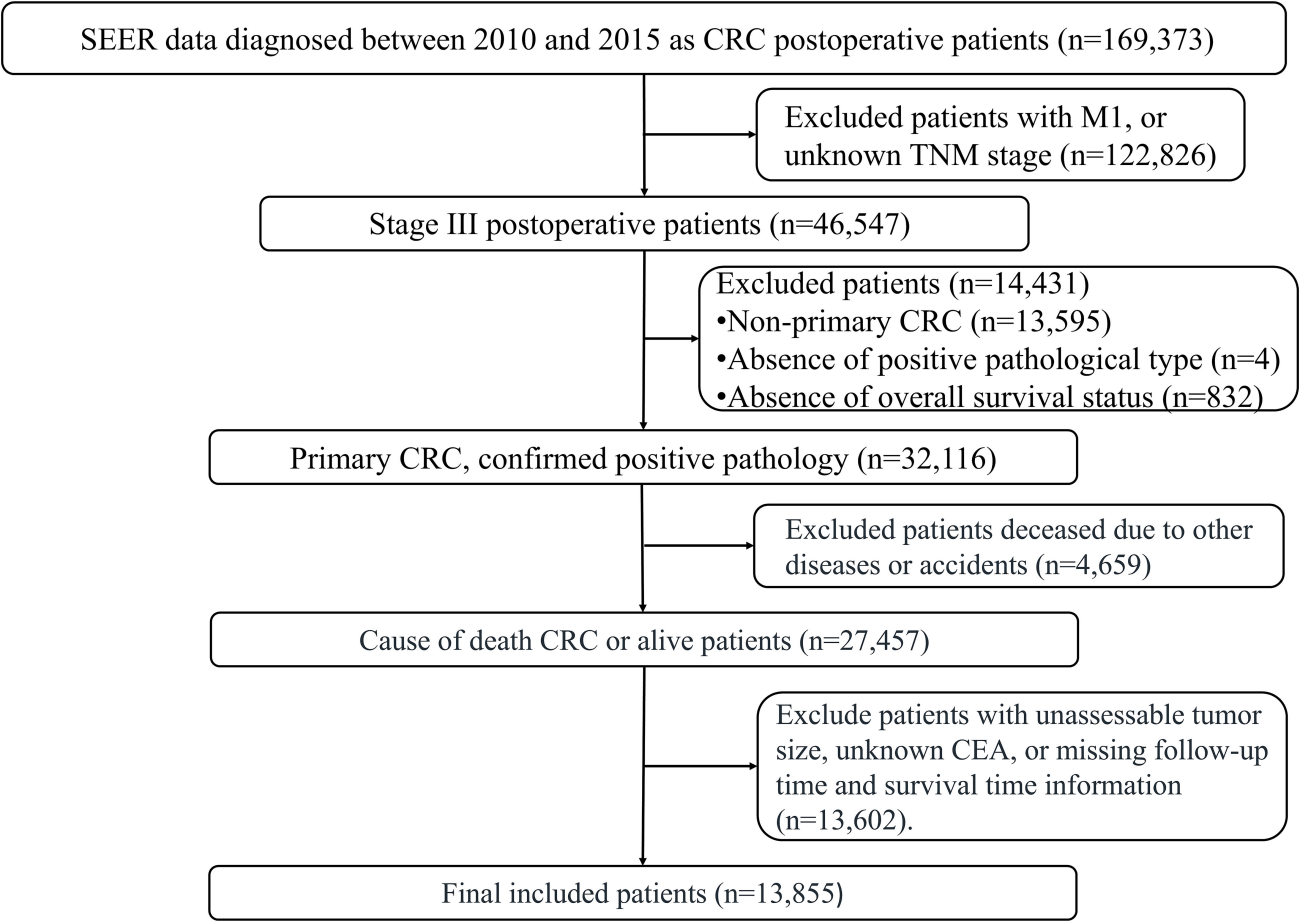
Patient selection flowchart from the SEER database.

**Figure 2:**
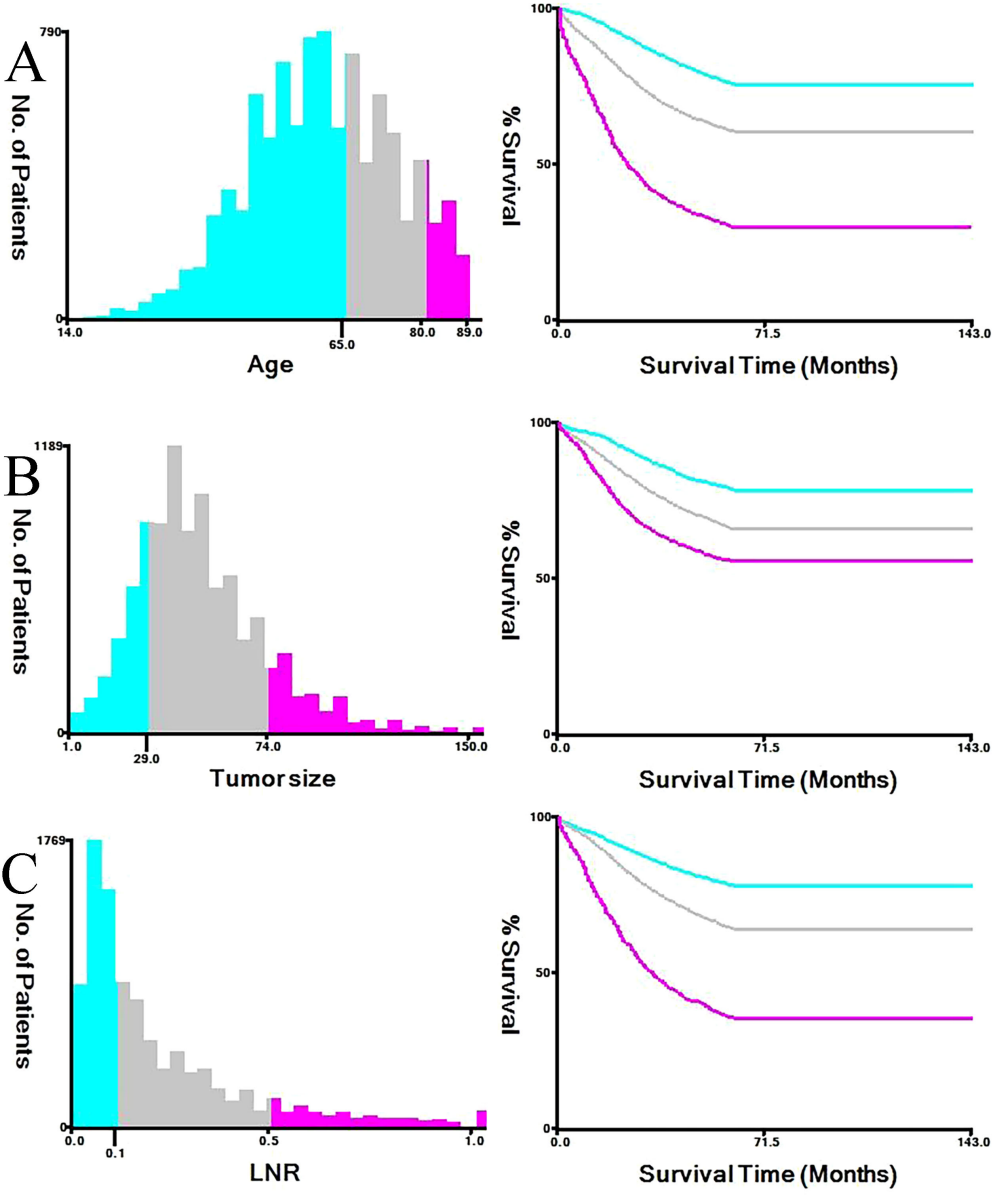
Optimal cutoff values for age **(A)**, maximum tumor diameter **(B)**, and lymph node ratio **(C)** determined by X-tile analysis.

After evaluating the missing data, we found that the missing rates for key variables, including chemotherapy status and CEA levels, were all below 5%. Therefore, incomplete records were excluded without imputation. Following the inclusion and exclusion criteria, a total of 13,855 patients with stage III colorectal cancer who underwent surgical treatment were included in the final analysis (see **Figure 1** for the detailed selection flowchart). The basic demographic characteristics of the study subjects are shown in **Table 1**. The subjects were then randomly divided into a training set (n = 9,699) and a validation set (n = 4,156) in a 7:3 ratio, with detailed data presented in **Table 2**. Thirteen prognostic-related variables were selected, including marital status, gender, tumor location, pathological type, T stage, radiotherapy and chemotherapy status, age, maximum tumor diameter, LNR, serum CEA levels, PNI, and tumor differentiation. No significant differences in demographic and clinical pathological characteristics were observed between the two groups (P > 0.05). The results from the X-tile software analysis indicated that the optimal cutoff points for age were 65 and 80 years, for maximum tumor diameter were 29 and 74 mm, and for LNR, they were 0.11 and 0.49 (see **Figure 2**).

**Table 1:** General information of patients.

Variable	Level	SEER cohort		External cohort		
		Survival	Death	Survival	Death
n		9171 (100.00)	4684 (100.00)	128 (100.00)	57 (100.00)
Marital status (%)	Married	5447 (59.39)	2301 (49.12)	117 (91.41)	45 (78.95)
	Unmarried	3330 (36.31)	2190 (46.75)	11 (8.59)	12 (21.05)
	Unknown	394 (4.30)	193 (4.12)	0 (0)	0 (0)
Sex (%)	Male	4675 (50.98)	2443 (52.16)	66 (51.56)	30 (52.63)
	Female	4496 (49.02)	2241 (47.84)	62 (48.44)	27 (47.37)
Tumor localization (%)	Right colon	3053 (33.29)	2032 (43.38)	49 (38.28)	17 (29.82)
	Transverse	533 (5.81)	355 (7.58)	1 (0.78)	0 (0.00)
	Left colon	759 (8.28)	350 (7.47)	10 (7.81)	7 (12.28)
	Rectosigmoid	3249 (35.43)	1219 (26.02)	27 (21.09)	9 (15.79)
	Rectum	1577 (17.20)	728 (15.54)	41 (32.03)	24 (42.11)
Pathological type (%)	Adenocarcinoma	8361 (91.17)	3957 (84.48)	117 (91.41)	50 (87.72)
	Not adenocarcinoma	810 (8.83)	727 (15.52)	11 (8.59)	7 (12.28)
T (%)	T1	449 (4.90)	38 (0.81)	11 (8.59)	0 (0.00)
	T2	1089 (11.87)	172 (3.67)	29 (22.66)	4 (7.02)
	T3	6281 (68.49)	2832 (60.46)	55 (42.97)	31 (54.39)
	T4	1352 (14.74)	1642 (35.06)	33 (25.78)	22 (38.60)
Radiation (%)	Yes	1681 (18.33)	728 (15.54)	1 (0.78)	2 (3.51)
	No	7490 (81.67)	3956 (84.46)	127 (99.22)	55 (96.49)
Chemotherapy (%)	Yes	7829 (85.37)	2910 (62.13)	109 (85.16)	38 (66.67)
	No	1342 (14.63)	1774 (37.87)	19 (14.84)	19 (33.33)
Age (%)	14-65	6336 (69.09)	2090 (44.62)	68 (53.12)	24 (42.11)
	66-80	2413 (26.31)	1637 (34.95)	52 (40.62)	24 (42.11)
	81-89	422 (4.60)	957 (20.43)	8 (6.25)	9 (15.79)
Tumor size (%)	1-29mm	1855 (20.23)	555 (11.85)	29 (22.66)	5 (8.77)
	30-74mm	6256 (68.22)	3287 (70.18)	89 (69.53)	41 (71.93)
	75-150mm	1060 (11.56)	842 (17.98)	10 (7.81)	11 (19.30)
LNR (%)	0.01-0.11	4515 (49.23)	1357 (28.97)	57 (44.53)	19 (33.33)
	0.12-0.49	4073 (44.41)	2311 (49.34)	59 (46.09)	27 (47.37)
	0.50-1.00	583 (6.36)	1016 (21.69)	12 (9.38)	11 (19.30)
CEA (%)	Positive	3340 (36.42)	2526 (53.93)	54 (42.19)	32 (56.14)
	Negative	5831 (63.58)	2158 (46.07)	74 (57.81)	25 (43.86)
PNI (%)	Yes	1380 (15.05)	1227 (26.20)	49 (38.28)	23 (40.35)
	No	7174 (78.22)	3061 (65.35)	79 (61.72)	34 (59.65)
	Unknown	617 (6.73)	396 (8.45)	0 (0)	0 (0)
Grade (%)	Grade I	508 (5.54)	170 (3.63)	19 (14.84)	5 (8.77)
	Grade II	6617 (72.15)	2737 (58.43)	84 (65.62)	36 (63.16)
	Grade III	1603 (17.48)	1368 (29.21)	24 (18.75)	15 (26.32)
	Grade IV	272 (2.97)	315 (6.73)	1 (0.78)	0 (0.00)
	Unknown	171 (1.86)	94 (2.01)	0 (0.00)	1 (1.75)

**Table 2:** Demographic information of stage III CRC postoperative patients in the training and validation cohorts.

Variable	Level	Total	Training set	Validation set	*P*
n		13855	9699	4156	
Marital status (%)	Married	7748 (55.92)	5446 (56.15)	2302 (55.39)	0.679
	Unmarried	5520 (39.84)	3841 (39.60)	1679 (40.40)	
	Unknown	587 (4.24)	412 (4.25)	175 (4.21)	
Sex (%)	Male	7118 (51.37)	4975 (51.29)	2143 (51.56)	0.771
	Female	6737 (48.63)	4724 (48.71)	2013 (48.44)	
Tumor localization (%)	Right colon	5085 (36.70)	3567 (36.78)	1518 (36.53)	0.611
	Transverse	888 (6.41)	640 (6.60)	248 (5.97)	
	Left colon	1109 (8.00)	782 (8.06)	327 (7.87)	
	Rectosigmoid	4468 (32.25)	3107 (32.03)	1361 (32.75)	
	Rectum	2305 (16.64)	1603 (16.53)	702 (16.89)	
Pathological type (%)	Adenocarcinoma	12318 (88.91)	8623 (88.91)	3695 (88.91)	0.998
	Not adenocarcinoma	1537 (11.09)	1076 (11.09)	461 (11.09)	
T (%)	T1	487 (3.51)	354 (3.65)	133 (3.20)	0.328
	T2	1261 (9.10)	863 (8.90)	398 (9.58)	
	T3	9113 (65.77)	6396 (65.94)	2717 (65.38)	
	T4	2994 (21.61)	2086 (21.51)	908 (21.85)	
Radiation (%)	Yes	2409 (17.39)	1671 (17.23)	738 (17.76)	0.452
	No	11446 (82.61)	8028 (82.77)	3418 (82.24)	
Chemotherapy (%)	Yes	10739 (77.51)	7513 (77.46)	3226 (77.62)	0.835
	No	3116 (22.49)	2186 (22.54)	930 (22.38)	
Age (%)	14-65	8426 (60.82)	5892 (60.75)	2534 (60.97)	0.935
	66-80	4050 (29.23)	2836 (29.24)	1214 (29.21)	
	81-89	1379 (9.95)	971 (10.01)	408 (9.82)	
Tumor size (%)	1–29 mm	2410 (17.39)	1669 (17.21)	741 (17.83)	0.295
	30–74 mm	9543 (68.88)	6672 (68.79)	2871 (69.08)	
	75–150 mm	1902 (13.73)	1358 (14.00)	544 (13.09)	
LNR (%)	0.01-0.11	5872 (42.38)	4105 (42.32)	1767 (42.52)	0.552
	0.12-0.49	6384 (46.08)	4456 (45.94)	1928 (46.39)	
	0.50-1.00	1599 (11.54)	1138 (11.73)	461 (11.09)	
CEA (%)	Positive	5866 (42.34)	4082 (42.09)	1784 (42.93)	0.360
	Negative	7989 (57.66)	5617 (57.91)	2372 (57.07)	
PNI (%)	Yes	2607 (18.82)	1798 (18.54)	809 (19.47)	0.367
	No	10235 (73.87)	7181 (74.04)	3054 (73.48)	
	Unknown	1013 (7.31)	720 (7.42)	293 (7.05)	
Grade (%)	Grade I	678 (4.89)	489 (5.04)	189 (4.55)	0.815
	Grade II	9354 (67.51)	6536 (67.39)	2818 (67.81)	
	Grade III	2971 (21.44)	2076 (21.40)	895 (21.54)	
	Grade IV	587 (4.24)	412 (4.25)	175 (4.21)	
	Unknown	265 (1.91)	186 (1.92)	79 (1.90)	

#### 3.1.2 External cohort patient data

In this study, 185 patients diagnosed with stage III CRC who underwent surgery at the General Surgery Department of Shanxi Bethune Hospital were selected to form the external validation cohort, in order to better evaluate the model’s performance in terms of discrimination, accuracy, and clinical applicability. As shown in **Table 1**, the patients included in our cohort were aged 14–65 years (92 patients), 66–80 years (76 patients), and 81–89 years (17 patients). Of these, 162 patients were married, and 23 were unmarried; 96 were male, and 89 were female. Regarding tumor location, 66 patients had tumors in the right colon, 1 in the transverse colon, 17 in the left colon, 36 at the rectosigmoid junction, and 65 in the rectum. The pathological types included 167 adenocarcinomas and 18 non-adenocarcinomas. For tumor staging, 11 patients were classified as T1, 33 as T2, 86 as T3, and 55 as T4. Three patients received adjuvant radiotherapy, and 147 received adjuvant chemotherapy. The maximum tumor diameter ranged from 1–29 mm (34 patients), 30–74 mm (130 patients), and 75–150 mm (21 patients). The LNR was 0.01-0.11 for 76 patients, 0.12-0.49 for 86 patients, and 0.50-1.00 for 23 patients. There were 86 patients with positive serum CEA levels (>5 ng/ml), 72 with PNI, and 113 without. Tumor differentiation grades included 24 well-differentiated tumors, 120 moderately differentiated tumors, 39 poorly differentiated tumors, 1 undifferentiated tumor, and 1 with unknown differentiation.

### 3.2 Factors influencing survival prognosis

**Figure 3:**
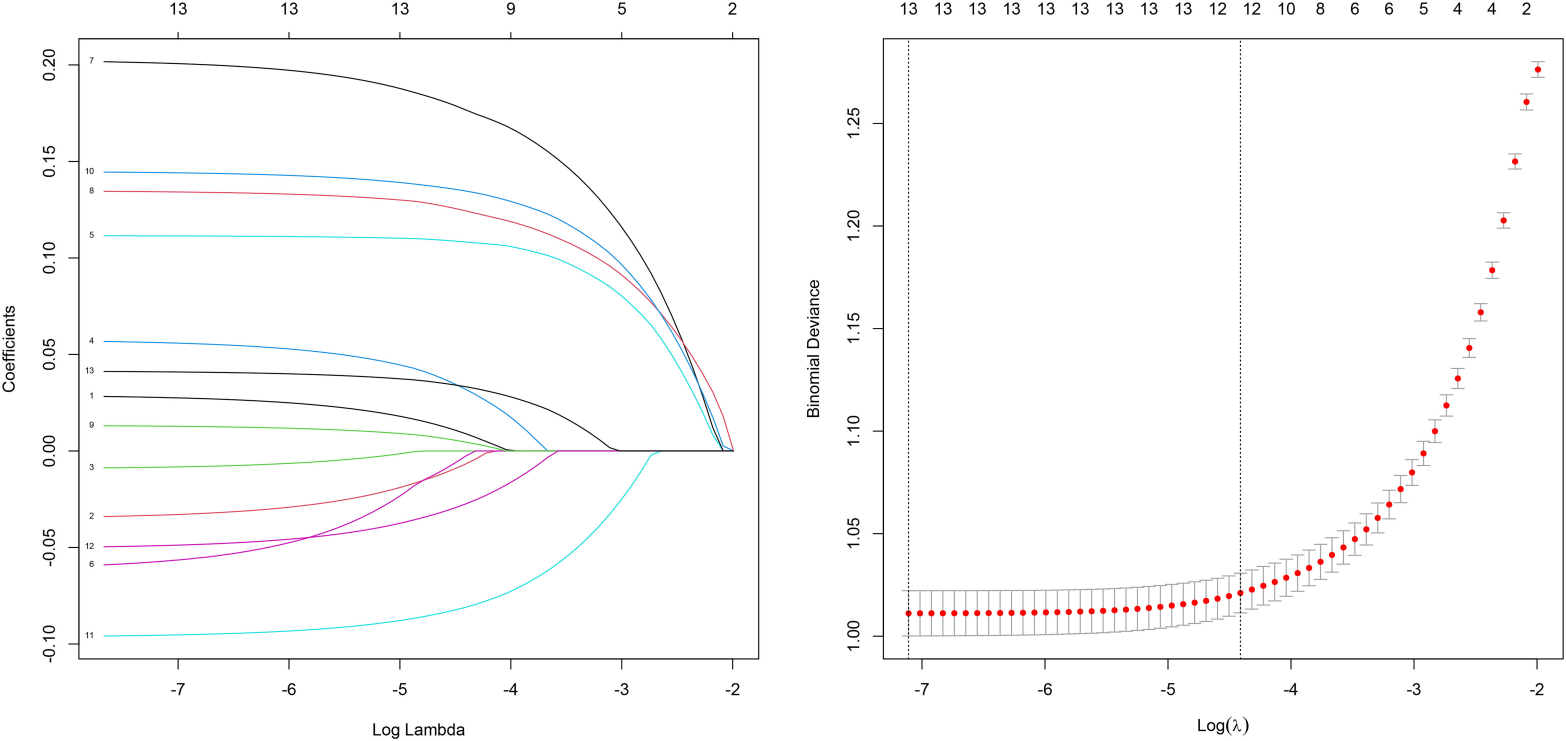
Lasso regression path plot.

Univariate logistic regression analysis revealed that marital status, gender, tumor location, pathological type, T stage, radiotherapy status, chemotherapy status, age, maximum tumor diameter, LNR, serum CEA levels, PNI, and tumor differentiation were significantly associated with the 5-year survival rate of stage III CRC postoperative patients (P < 0.05). **Supplementary Table 1** presents the specific assignment method for the independent variables, and **Table 3** shows the analysis data for factors affecting the postoperative survival prognosis of stage III CRC patients. Factors with statistical significance were included in a multivariate logistic regression analysis using a backward stepwise method. The results indicated that general characteristics (unmarried, age > 65 years), tumor pathological features (non-adenocarcinoma, T stage T1 or T3, maximum tumor diameter > 75 mm, LNR > 0.11, poorly differentiated or undifferentiated tumors) were independent risk factors for postoperative survival in stage III CRC patients (P < 0.05). On the other hand, tumor location at the rectosigmoid junction, negative serum CEA levels, and the absence or unknown status of perineural invasion were independent protective factors for postoperative survival in stage III CRC patients (P < 0.05). The variables selected by Lasso regression were consistent with those identified by multivariate logistic regression, including marital status, age, T stage, maximum tumor diameter, lymph node ratio (LNR), and tumor differentiation. The Lasso coefficient path plot (**Figure 3**) illustrates the changes in variable coefficients as the regularization parameter lambda varies. Through regularization, Lasso effectively selected variables significantly associated with survival status, ensuring the simplicity and stability of the final model. Using both VIF analysis and correlation tests between variables, the results showed that the VIF for all explanatory variables did not exceed the critical value of 10, and the Pearson correlation coefficients between variables remained below 0.8, confirming the absence of significant multicollinearity in the model (see **Supplementary Figures 1**, **2**).

**Table 3:** Univariate and multivariate logistic analysis of prognosis in stage III CRC postoperative patients.

Variable	Level	Univariate analysis			Multivariate analysis		
		OR	95%CI	*P*	OR	95%CI	*P*
Marital status	Married	Ref			Ref		
	Unmarried	1.579	1.448-1.721	**<0.001**	1.274	1.150-1.410	**<0.001**
	Unknown	1.118	0.901-1.387	0.312	0.953	0.738-1.229	0.709
Sex	Male	Ref			Ref		
	Female	0.952	0.875-1.036	0.257	–	–	–
Tumor localization	Right colon	Ref			Ref		
	Transverse	0.947	0.797-1.126	0.540	1.038	0.847-1.273	0.720
	Left colon	0.701	0.594-0.826	**<0.001**	0.827	0.681-1.004	0.055
	Rectosigmoid	0.567	0.511-0.629	**<0.001**	0.796	0.702-0.902	**<0.001**
	Rectum	0.696	0.614-0.789	**<0.001**	1.048	0.849-1.292	0.665
Pathological type	Adenocarcinoma	Ref			Ref		
	Not adenocarcinoma	1.885	1.659-2.141	**<0.001**	1.318	1.129-1.538	**<0.001**
T	T1	Ref			Ref		
	T2	1.727	1.133-2.633	**0.011**	1.337	0.850-2.103	0.209
	T3	4.793	3.283-6.997	**<0.001**	2.735	1.808-4.135	**<0.001**
	T4	13.116	8.932-19.259	**<0.001**	6.275	4.109-9.582	**<0.001**
Radiation	Yes	Ref			Ref		
	No	1.177	1.051-1.319	**0.005**	0.787	0.648-0.955	**0.016**
Chemotherapy	Yes	Ref			Ref		
	No	3.640	3.300-4.015	**<0.001**	2.782	2.458-3.147	**<0.001**
Age	14-65	Ref			Ref		
	66-80	2.038	1.851-2.243	**<0.001**	1.802	1.611-2.015	**<0.001**
	81-89	7.214	6.215-8.372	**<0.001**	4.657	3.896-5.566	**<0.001**
Tumor size	1-29mm	Ref			Ref		
	30-74mm	1.866	1.643-2.119	**<0.001**	1.134	0.975-1.319	0.100
	75-150mm	2.870	2.449-3.364	**<0.001**	1.239	1.025-1.499	**0.028**
LNR	0.01-0.11	Ref			Ref		
	0.12-0.49	1.978	1.797-2.177	**<0.001**	1.975	1.774-2.200	**<0.001**
	0.50-1.00	6.401	5.558-7.371	**<0.001**	5.317	4.528-6.244	**<0.001**
CEA	Positive	Ref			Ref		
	Negative	0.474	0.435-0.517	**<0.001**	0.582	0.526-0.643	**<0.001**
PNI	Yes	Ref			Ref		
	No	0.472	0.425-0.525	**<0.001**	0.606	0.536-0.686	**<0.001**
	Unknown	0.763	0.641-0.909	**0.003**	0.744	0.605-0.914	**0.005**
Grade	Grade I	Ref			Ref		
	Grade II	1.178	0.955-1.453	0.126	1.104	0.871-1.399	0.416
	Grade III	2.515	2.015-3.138	**<0.001**	1.474	1.144-1.897	**0.003**
	Grade IV	3.369	2.546-4.459	**<0.001**	1.999	1.447-2.763	**<0.001**
	Unknown	1.678	1.17-2.407	**0.005**	1.321	0.872-2.001	0.190

“-” indicates no data, bold indicates P < 0.05.

### 3.3 Construction of nomogram

**Figure 4:**
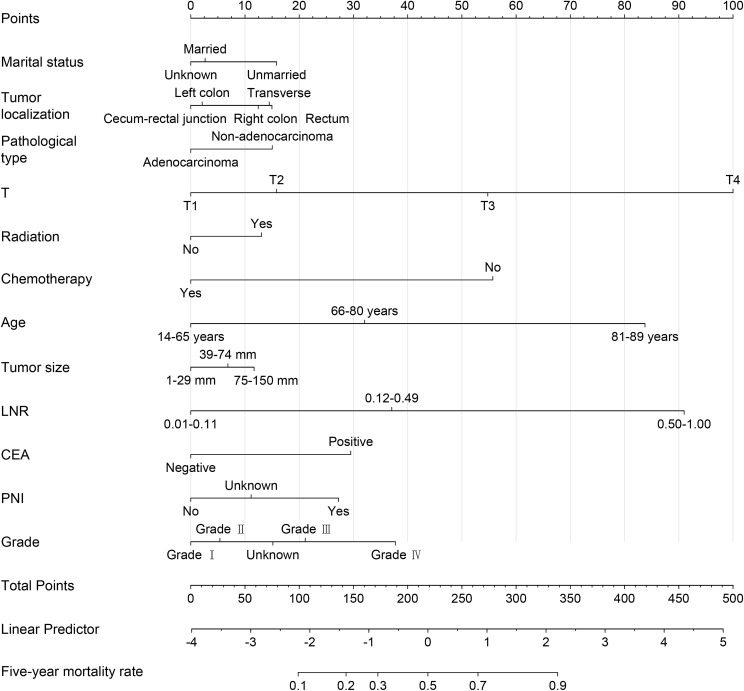
Nomogram.

A nomogram was constructed based on the results of the multivariate logistic regression analysis, as shown in **Figure 4**. By calculating the score for each factor, the 5-year mortality risk for stage III CRC postoperative patients can be predicted. The dynamic nomogram for predicting the 5-year survival status of stage III CRC postoperative patients is available at https://zhangwei2530.shinyapps.io/DynNomapp001/.

### 3.4 Machine learning algorithms

**Figure 5:**
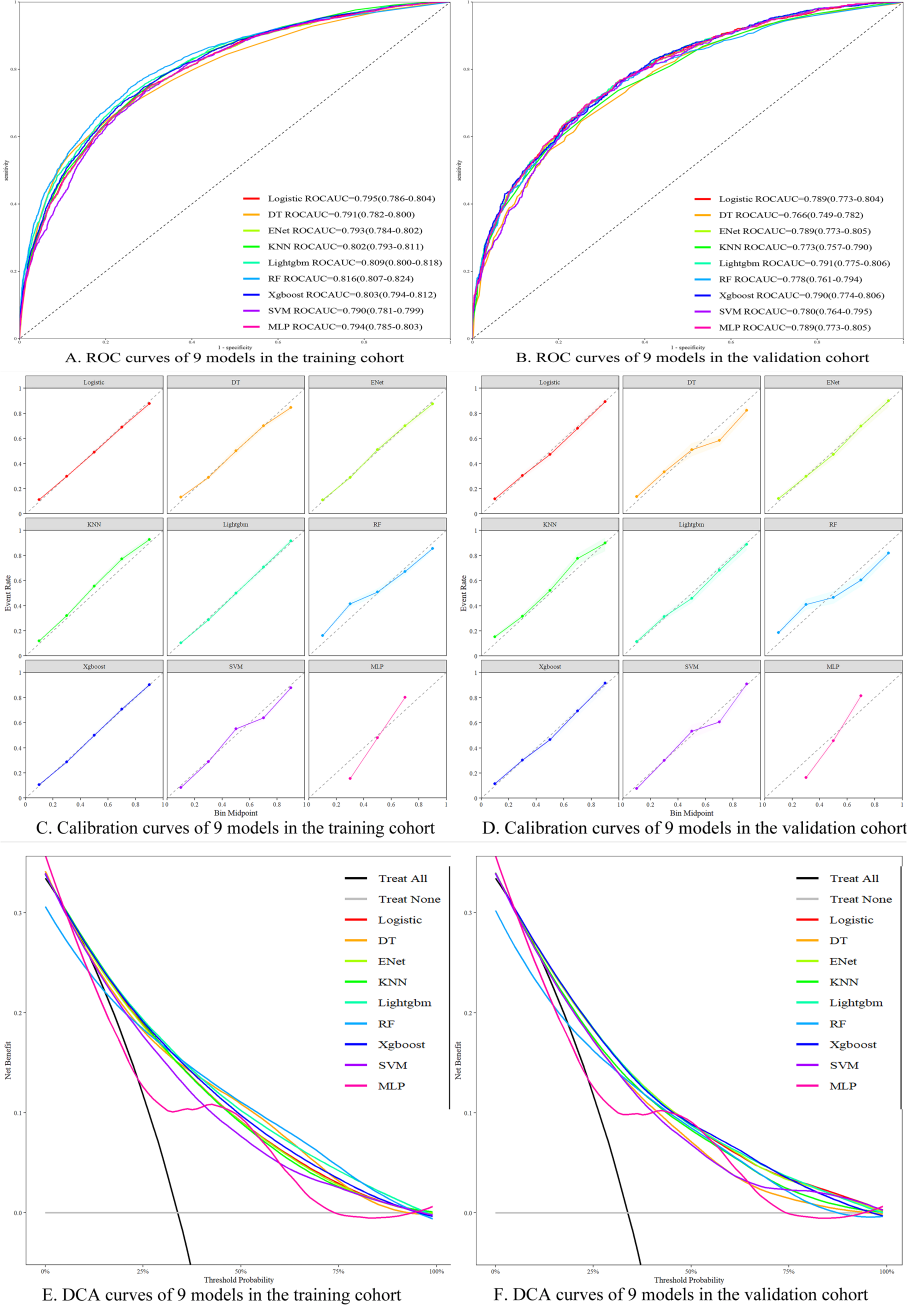
Performance evaluation of different machine learning models. **(A)** ROC curves of each model in the training set; **(B)** ROC curves of each model in the validation set; **(C)** Calibration curves of each model in the training set; **(D)** Calibration curves of each model in the validation set; **(E)** DCA curves of each model in the training set; **(F)** DCA curves of each model in the validation set.

To ensure the accuracy of our nomogram prediction model, new machine learning algorithms were applied to the validation set (n = 4,714). The prediction performance of the nine models in both the training and validation sets is shown in **Table 4**. The ROC curves for the nine models in both the training and validation sets are depicted in **Figures 5A, B**. Subsequently, calibration plots for the nine models were constructed, as shown in **Figures 5C, D**. The calibration curves for the 5-year survival prognosis of stage III CRC postoperative patients are close to the ideal 45° line, indicating that algorithms, including Logistic regression and LightGBM, have predictive value. Among these, the LightGBM model showed the strongest consistency with the observed outcomes for predicting the 5-year survival prognosis. The DCA plots (**Figures 5E, F**) demonstrate that all nine models provide a good clinical net benefit. We found that the Logistic regression-based prediction model achieved an ROC area of 0.789 in the validation set, with strong performance across various prediction metrics, indicating that the nomogram has robust predictive ability and clinical applicability. The LightGBM model achieved an ROC area of 0.791 in the validation set, demonstrating strong classification ability. Its precision was 0.570, and its recall was 0.686, showing balanced precision and recall, indicating good accuracy and strong recall capacity, thus avoiding bias toward any particular class. Additionally, LightGBM demonstrated efficiency in handling large-scale datasets, with faster training speed and lower memory consumption, making it suitable for complex classification tasks. In comparison, other models such as XGBoost and RF also performed well, but LightGBM outperforms in terms of overall performance, training efficiency, and data processing capability. Therefore, LightGBM is considered the optimal model.

**Table 4:** Predictive performance of nine models in the training and validation cohorts.

Dataset	Model	Accuracy	Precision	Recall	F1 Score	ROC
Training cohort	LR	0.725	0.573	0.730	0.642	0.795
	DT	0.744	0.613	0.658	0.635	0.791
	ENet	0.725	0.574	0.718	0.638	0.793
	KNN	0.714	0.556	0.762	0.643	0.802
	LightGBM	0.740	0.595	0.721	0.652	0.809
	RF	0.743	0.596	0.747	0.663	0.816
	XGboost	0.739	0.596	0.708	0.647	0.803
	SVM	0.717	0.562	0.735	0.637	0.790
	MLP	0.719	0.564	0.740	0.640	0.794
Validation cohort	LR	0.706	0.550	0.712	0.621	0.789
	DT	0.721	0.585	0.602	0.593	0.766
	ENet	0.712	0.559	0.711	0.626	0.789
	KNN	0.687	0.527	0.737	0.614	0.773
	LightGBM	0.719	0.570	0.686	0.622	0.791
	RF	0.707	0.552	0.711	0.621	0.778
	XGboost	0.715	0.565	0.678	0.616	0.790
	SVM	0.702	0.544	0.729	0.623	0.779
	MLP	0.703	0.545	0.730	0.624	0.789

### 3.5 SHAP model interpretation

**Figure 6:**
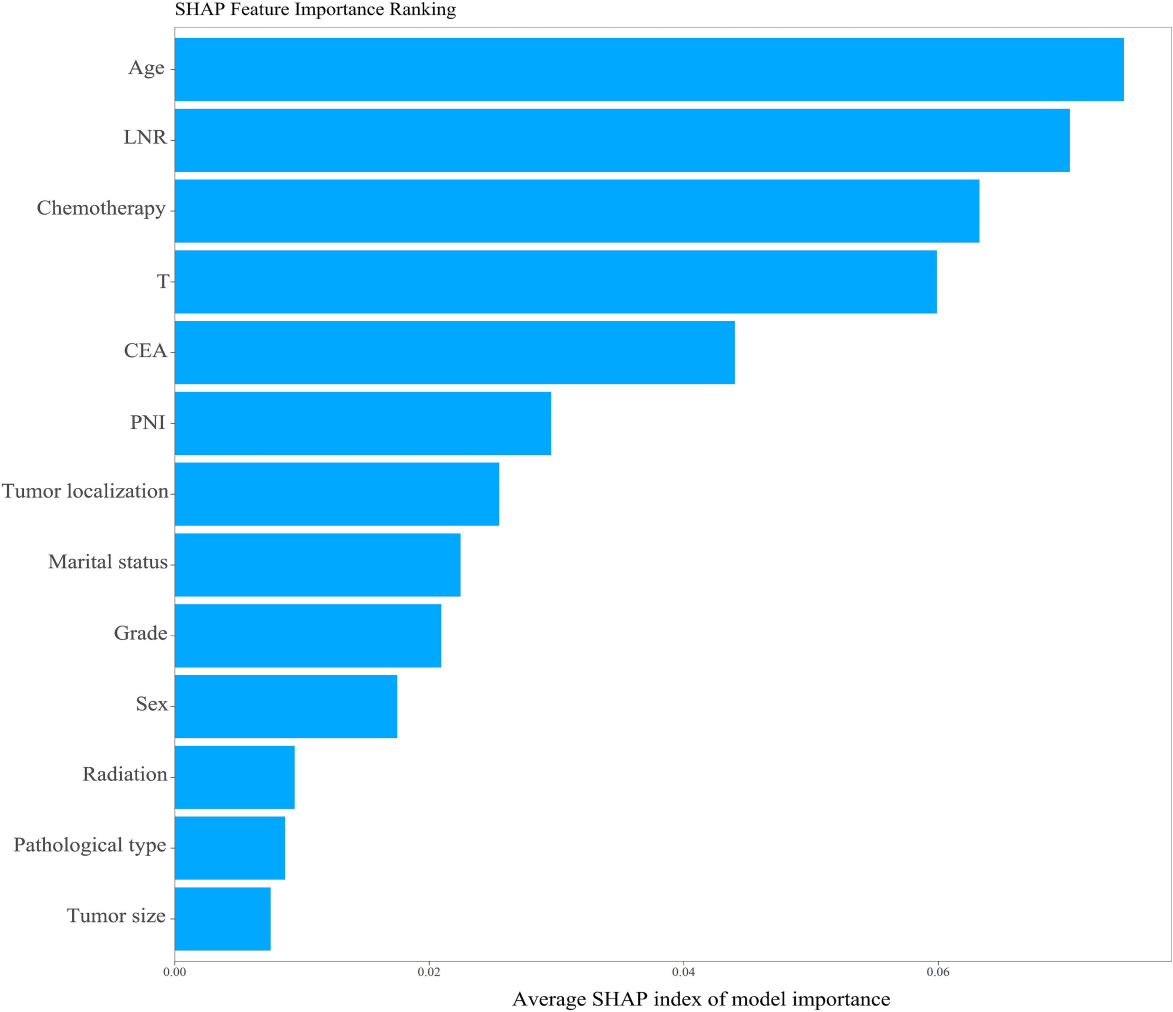
Feature importance ranking based on the LightGBM model.

**Figure 7:**
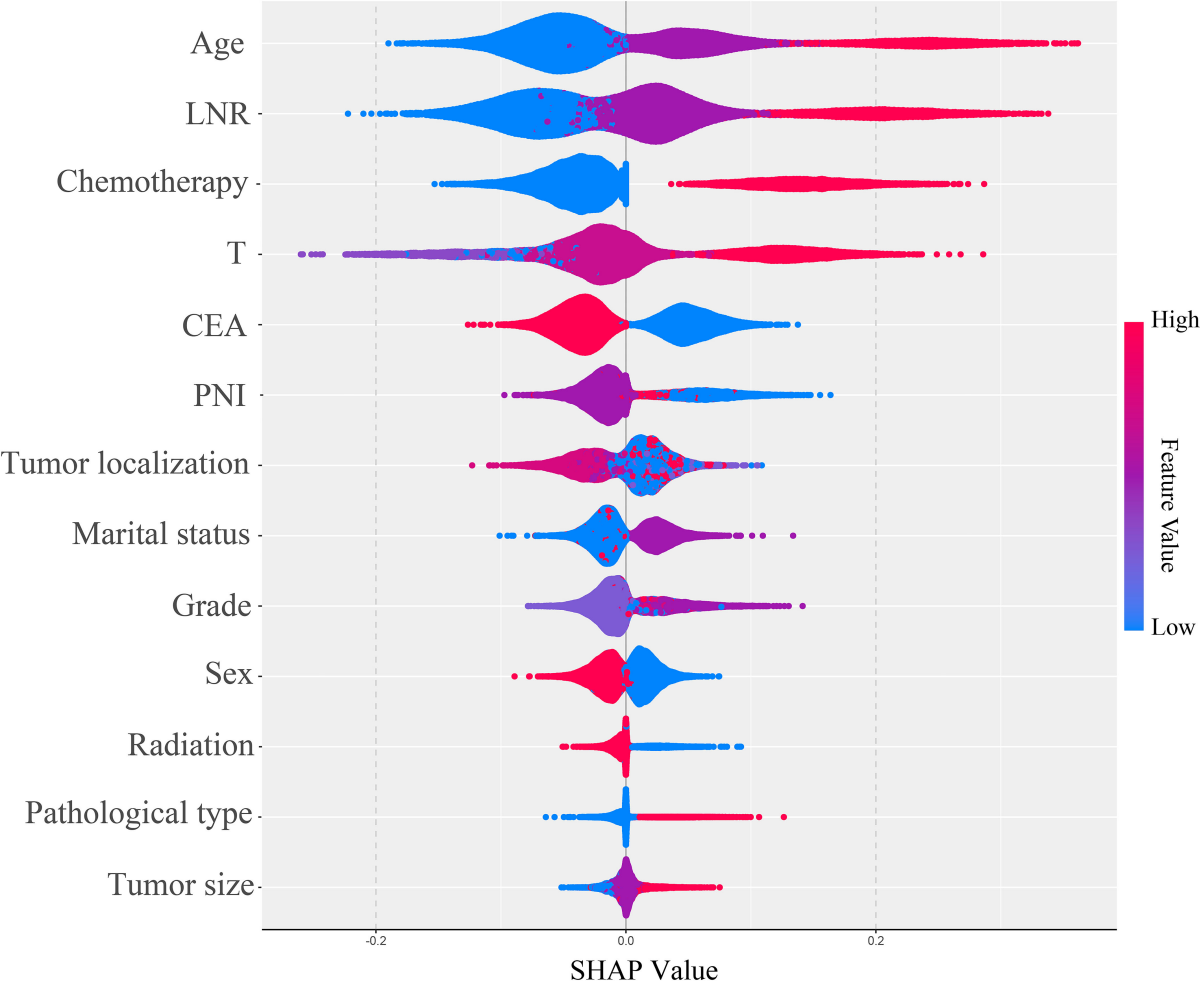
SHAP summary plot for the LightGBM model.

Based on the SHAP feature importance analysis of the optimal LightGBM model, age, LNR, chemotherapy status, and T stage were identified as the most important factors for predicting the cancer-specific survival status of stage III CRC postoperative patients (as shown in **Figure 6**). Higher age, higher LNR, lack of chemotherapy, higher T stage, positive serum CEA, perineural invasion, radiotherapy, adenocarcinoma, and larger maximum tumor diameter all had a positive impact on the model, increasing the risk of death within 5 years. In contrast, marital status had a negative impact on the model and was considered a protective factor for postoperative survival in stage III CRC patients. Patients with tumors located in the right colon and rectum had a higher risk of death compared to those with tumors in the left colon. Additionally, male patients had a higher risk of death than female patients, and adenocarcinoma patients had a higher risk of death compared to non-adenocarcinoma patients (see **Figure 7**).

**Figure 8:**
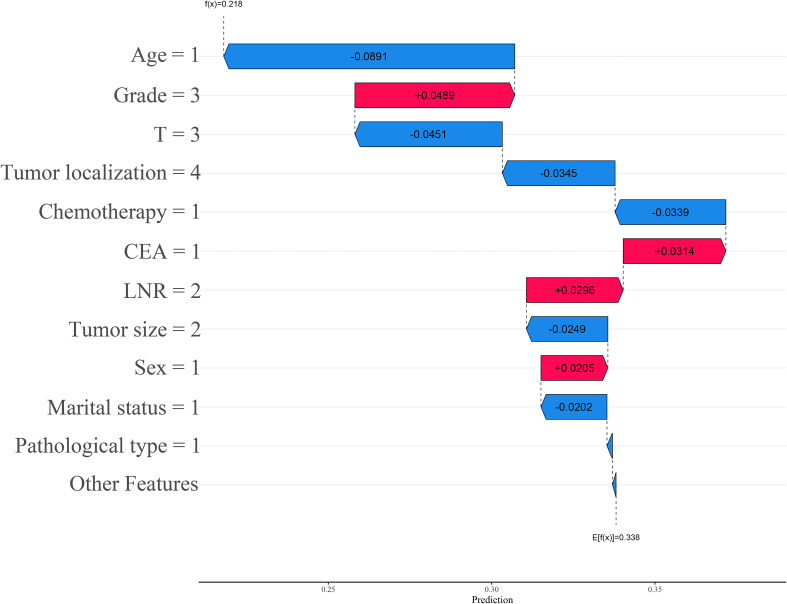
Interactive SHAP plot for surviving patients.

**Figure 9:**
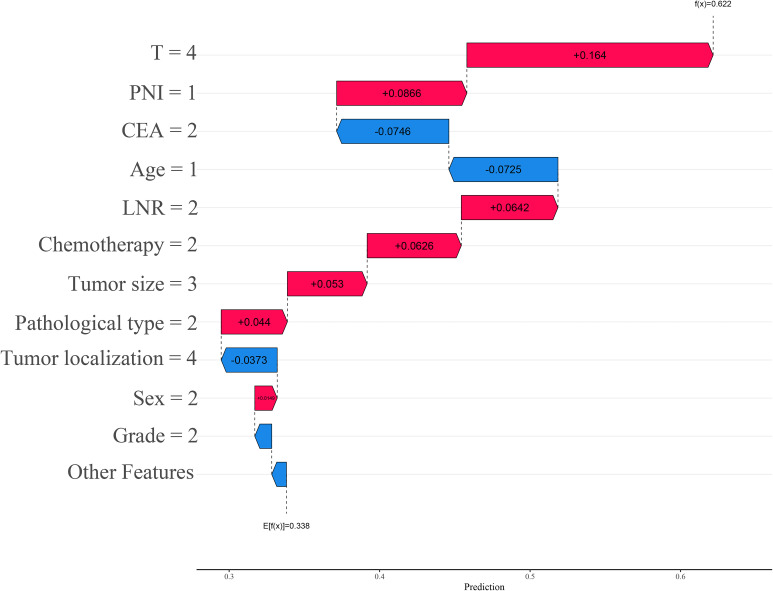
Interactive SHAP plot for deceased patients.

The interactive SHAP plot provides a visualization of the selected individual patient from the randomly extracted data. In the plot, the red portion indicates a positive contribution to the risk of death, while the blue portion indicates a negative contribution to survival. Arrows pointing left represent a decrease in SHAP value, while arrows pointing right represent an increase, with longer arrows indicating a greater contribution of that feature to the final outcome. For surviving patients, the interactive SHAP plot shows that age ≤ 65 years, T stage T3, tumor located at the rectosigmoid junction, a history of chemotherapy, maximum tumor diameter between 30–74 mm, and marital status were all protective factors for survival, with the final SHAP value of 0.218 (see **Figure 8**). In contrast, for deceased patients, the interactive SHAP plot revealed that T stage T4, perineural invasion, LNR between 0.12 and 0.49, lack of chemotherapy, maximum tumor diameter between 75–150 mm, non-adenocarcinoma pathological type, and female gender were promoting factors for death, with the final SHAP value of 0.622 (see **Figure 9**). To further understand the potential limitations of the model in clinical applications, we conducted a detailed analysis of the confusion matrix of the LightGBM model in the validation set. The results showed 665 true positives, 332 false positives, 760 false negatives, and 2,399 true negatives. The false positive rate was 12.2%, and the false negative rate was 24.1%. These misclassified cases provide valuable additional insight into the evaluation of the model’s performance.

### 3.6 Survival analysis

**Figure 10:**
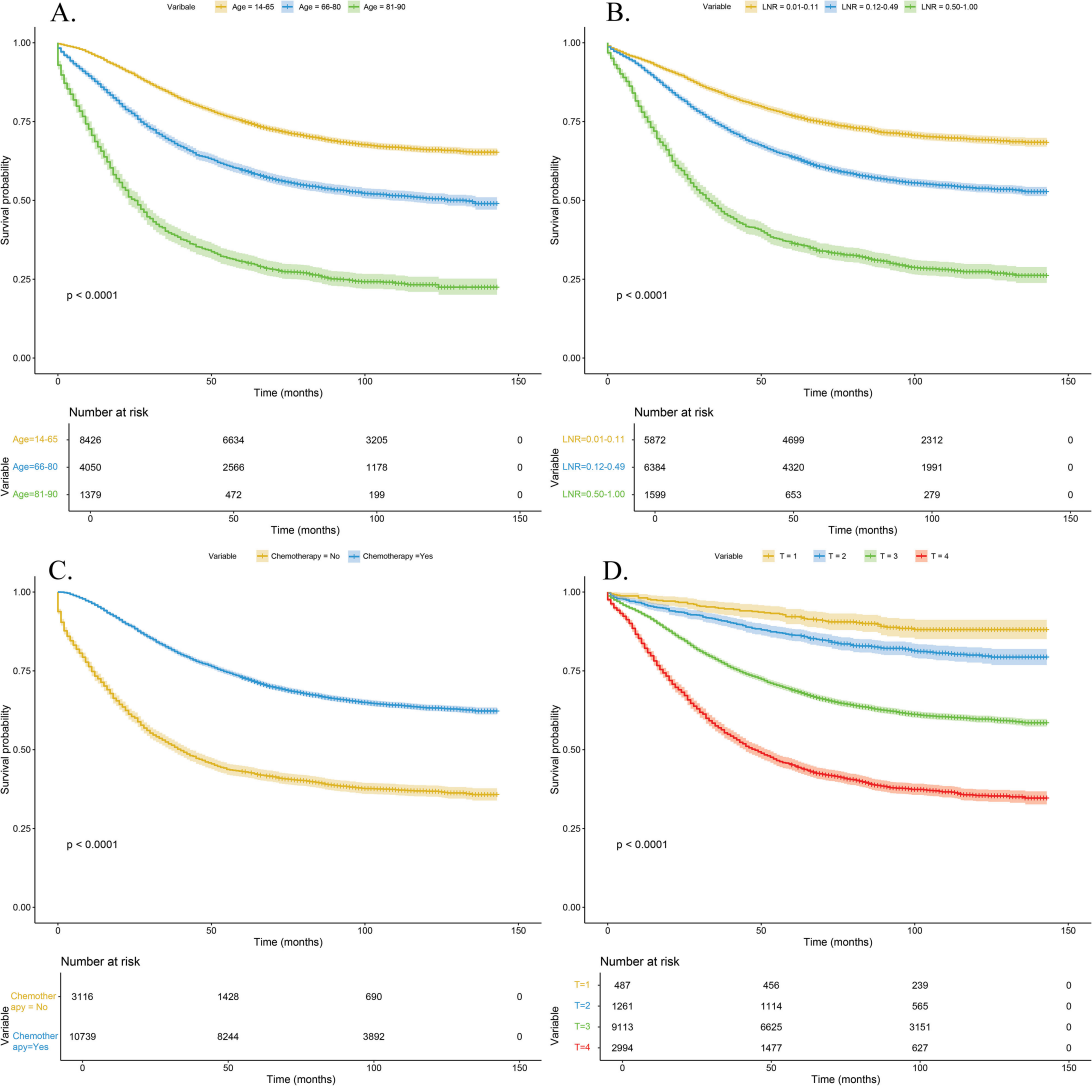
Kaplan-Meier survival curves for different clinical subgroups. **(A)** Survival curves by age group; **(B)** Survival curves by LNR levels; **(C)** Survival curves by chemotherapy status; **(D)** Survival curves by T stage.

In the survival analysis, we used the Kaplan-Meier method to evaluate the 5-year cancer-specific survival of patients with stage III colorectal cancer, stratified by LNR, T stage, age group, and chemotherapy status (see **Figure 10**). The results showed that the survival curves differed significantly among the variable groups (Log-rank test, *P* < 0.05).

### 3.7 External validation

**Figure 11:**
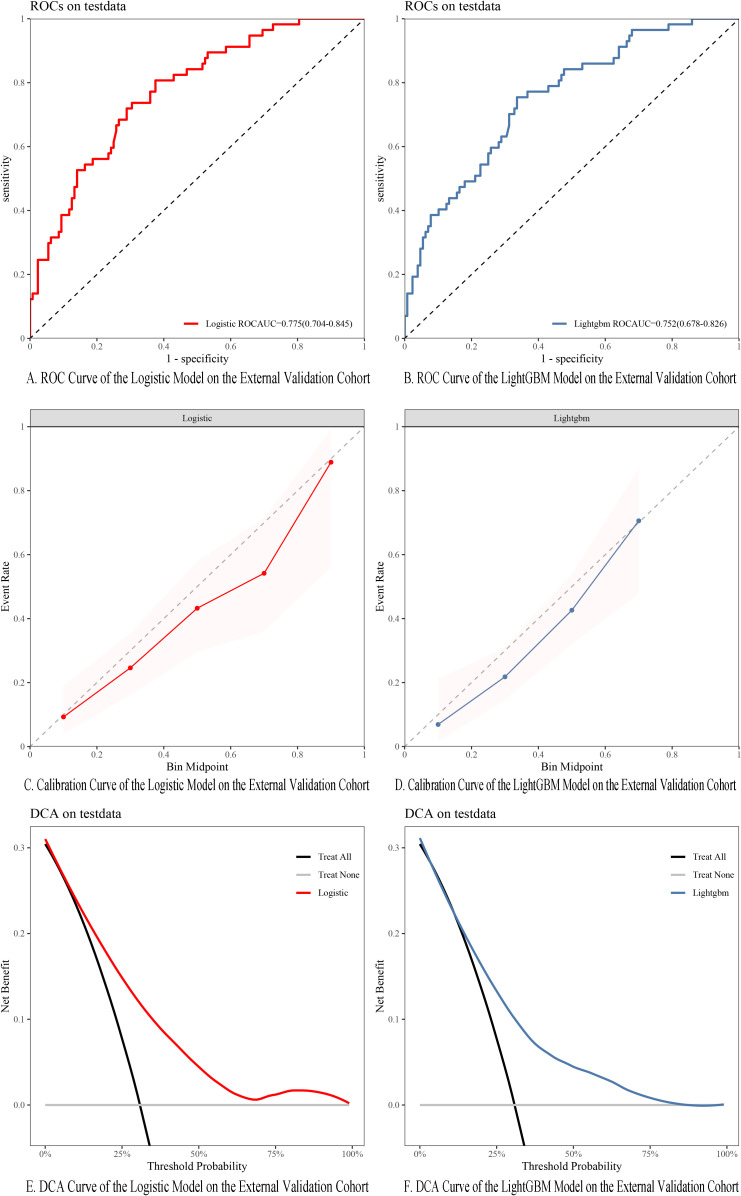
**(A)** ROC curve of Logistic model in external validation cohort. **(B)** ROC curve of LightGBM model in external validation cohort. **(C)** Calibration curves of the Logistic models in the external validation cohort. **(D)** Calibration curves of the LightGBM models in the external validation cohort. **(E)** DCA of the Logistic models in the external validation cohort. **(F)** DCA of the LightGBM models in the external validation cohort.

In the external validation cohort, the AUC for the Logistic regression model and LightGBM model reached 0.775 and 0.752, respectively (**Figures 11A, B**), indicating that both the nomogram based on the Logistic regression model and the SHAP model explanation based on the LightGBM model exhibit strong predictive ability. Calibration curve analysis showed that the predicted probabilities were in good agreement with the actual observed values, with both closely aligning near the 45° reference line (**Figures 11C, D**). The DCA curve further confirmed the clinical applicability of both models (**Figures 11E, F**).

## 4 Discussion

CRC is one of the most prevalent malignant tumors globally, with its mortality rate ranking among the highest across various types of cancer. In recent years, the incidence and mortality rates of this disease have steadily increased due to changes in lifestyle and dietary patterns (2). Stage III CRC refers to tumors that have spread to nearby lymph nodes but have not yet metastasized to distant sites. Among stage III CRC patients, there is considerable variability in the five-year postoperative survival rates, which are closely associated with the patient’s general clinical characteristics, pathological features, and treatment methods. Although the current TNM staging system provides a fundamental framework for cancer prognosis, it does not fully account for all factors that may affect patients’ survival. Therefore, prognostic prediction models based on individualized factors, particularly those constructed using machine learning (ML) methods, are essential for improving the accuracy of predicting the five-year survival status of stage III CRC patients.

This study developed a five-year survival prediction model for stage III CRC patients using machine learning (ML) methods based on the SEER database and conducted external validation. It also investigated the impact of various clinical and pathological factors on patient prognosis. Multivariate regression analysis revealed that several clinical and pathological factors were independent predictors of prognosis in stage III CRC patients. We found that general characteristics (unmarried, age over 65), tumor pathological features (non-adenocarcinoma, T stage T1 or T3, maximum tumor diameter greater than 63 mm, LNR greater than 0.11, poorly or undifferentiated tumors) were associated with poorer postoperative survival prognosis. Conversely, tumor location at the rectosigmoid junction, negative serum CEA levels, and the absence or unknown status of perineural invasion were identified as independent protective factors for postoperative survival in stage III CRC patients. In this study, a Kaplan-Meier analysis was performed to assess the 5-year cancer-specific survival of patients. The results showed significant survival differences across subgroups defined by LNR, T stage, age, and chemotherapy status. These findings were highly consistent with the key variables identified by the model, further supporting the clinical relevance of the model’s interpretability. Specifically, patients with high LNR, advanced T stage, older age, and those who did not receive chemotherapy exhibited significantly lower 5-year survival rates. These factors can serve as important references for clinical risk assessment. The results further underscore the prognostic value of LNR as a more sensitive indicator than the traditional N stage. Additionally, the Kaplan-Meier curves clearly illustrated the substantial impact of chemotherapy in prolonging survival, highlighting the importance of postoperative intervention and management in high-risk individuals. Future research may build on these findings to develop refined risk stratification strategies and assist in formulating personalized treatment and follow-up plans to improve survival outcomes in patients with stage III colorectal cancer.

Several studies have confirmed that advanced age and unmarried status are independent risk factors for CRC (15), a conclusion consistent with the data from this study and the SHAP model interpretability analysis. The poorer prognosis observed in elderly patients may be attributed to a combination of physiological and psychological factors. From a physiological perspective, aging is often accompanied by multiple chronic conditions such as cardiovascular disease and diabetes, along with declining organ function and weakened immune responses. These factors can reduce tolerance to surgery and chemotherapy, thereby limiting treatment intensity and efficacy. Additionally, tumors in older individuals may exhibit greater molecular heterogeneity and more aggressive phenotypes, further impacting overall survival. On the psychological level, elderly patients are more prone to anxiety, depression, and other negative emotions, which can reduce treatment adherence and hinder postoperative recovery. Unmarried status may also adversely affect treatment outcomes due to a lack of social support. Studies have shown that spouses can provide emotional comfort, daily care, and decision-making support throughout the treatment process, thereby improving patient compliance and quality of life (16). In contrast, unmarried patients often experience loneliness and a lack of caregiving resources, which can lead to psychological distress and treatment delays. The absence of intimate relationships may also diminish patients’ motivation to engage in healthy behaviors, affecting follow-up care and access to secondary treatments (17, 18). Taken together, these factors contribute to the identification of advanced age and unmarried status as adverse prognostic indicators. Future interventions should focus on providing comprehensive support for these high-risk groups, such as implementing geriatric oncology multidisciplinary team (MDT) management, enhancing psychological care, and strengthening social support networks, in order to improve long-term outcomes through holistic patient management.

Adenocarcinoma is the dominant histological subtype of CRC, accounting for more than 90% of all cases. Clinical observations show that adenocarcinoma patients have a clear survival advantage, particularly when compared with subtypes such as mucinous adenocarcinoma and signet-ring cell carcinoma, where non-adenocarcinoma patients exhibit significantly poorer survival curves. Our study confirms this, finding that non-adenocarcinoma types are associated with poorer survival prognosis, further supported by the SHAP model interpretation. Research by Hugen N et al. (19) also verified this, showing that mucinous adenocarcinoma and signet-ring cell carcinoma cases not only exhibit increased metastasis rates but also demonstrate a tendency for multi-organ metastasis. This clinical phenomenon is closely related to the unique molecular biological characteristics and invasive metastatic mechanisms of these tumor types. Pathologically, mucinous adenocarcinoma is characterized by a significant increase in mucin production within the tumor area. These mucinous components not only cause characteristic histological changes but also provide a material foundation for tumor cells to disseminate through the vascular system. Signet-ring cell carcinoma cells exhibit a typical “ring” shape, which directly enhances their ability to disrupt the extracellular matrix. After breaking through local tissue barriers, these cells metastasize to distant organs via the circulatory system.

The AJCC TNM staging system, as the gold standard for CRC clinical diagnosis and treatment, is valuable for systematically evaluating tumor invasion depth, lymph node involvement, and distant metastasis. Tumor infiltration depth is positively correlated with invasive and metastatic potential, and when the tumor penetrates from the muscularis propria to the subserosal layer, the potential for lymph node metastasis increases exponentially. Based on our findings, both T1 and T3 are independent risk factors for CRC. The SHAP model interpretability results indicate that T4 stage significantly influences prognosis, increasing the risk of death within five years. The association between tumor size and CRC survival outcomes has been supported by several cohort studies (6, 20). Larger malignant tumors often present with more aggressive phenotypes and metastatic potential, with a clear causal relationship to poor clinical outcomes. When tumor size exceeds a critical threshold, it often suggests an increased likelihood of local tissue infiltration or lymphatic involvement, leading to an elevated risk of disease progression. Our data analysis further confirms the clinical value of tumor size as an independent prognostic marker, highlighting its significance in guiding the development of individualized treatment plans.

Compared to traditional qualitative lymph node positivity/negativity assessments and the quantitative evaluation of the number of positive lymph nodes in N staging, the LNR has shown significant superiority in depicting tumor biological characteristics and predicting patient survival prognosis. Research indicates that LNR holds substantial medical application value in predicting the survival of patients with digestive system malignancies. Relevant literature has validated its effectiveness in prognosis evaluation for esophageal cancer (21), pancreatic cancer (22), and gastric cancer (23). Our study also confirms this, finding that an LNR greater than 0.11 is an independent risk factor for CRC. Additionally, the SHAP model interpretation further supports this finding, showing that when LNR exceeds 0.11, it positively contributes to the model and increases the 5-year mortality risk for patients. Ceelen et al. (24), through a systematic review of 16 studies, recommend using 0.10 as the threshold for positive lymph nodes, which is consistent with our findings.

Studies have shown that poorly or undifferentiated tumor states are closely associated with poor postoperative prognosis in stage III CRC patients. This conclusion has been validated in similar studies (25), including one cohort study involving 391 surgical cases, where tumor differentiation was identified as an independent risk factor affecting patient survival prognosis. Tumor differentiation is generally considered an important indicator in cancer prognosis evaluation. Poorly differentiated cancer cells are characterized by high malignancy, rapid growth, and strong invasive abilities. These cases are prone to early local breakthrough and distant metastasis, directly leading to worsened clinical outcomes. Poorly or undifferentiated tumor cells often lack the structural characteristics of normal cells, making the tumor more likely to break through local barriers early and extend to adjacent tissues or distant organs, thus adversely affecting patients’ survival.

In the survival analysis of stage III CRC patients, differences in tumor primary site significantly impacted prognosis. Patients with left-sided colon cancer had notably higher survival rates than those with right-sided colon or rectal cancer. This disparity may arise from biological differentiation due to different embryonic origins; the left colon originates from the hindgut, while the right colon is derived from the midgut. Key molecular mechanisms include microsatellite instability (MSI), BRCA mutations, and the KRAS signaling pathway (26). Tumors in the right colon often exhibit an MSI-H phenotype, while KRAS mutations are predominant in the left colon. These molecular differences may contribute to the varying sensitivity of tumors located in different anatomical sites to chemotherapy. Clinical data show that patients with left-sided colon cancer who receive standard chemotherapy regimens have a total survival time 4–6 months longer than those with right-sided colon cancer. Notably, while rectal cancer also originates from the hindgut, its unique anatomical location leads to differences in lymphatic drainage pathways, which may explain why the survival curves of rectal cancer patients do not significantly converge with those of left-sided colon cancer patients. Molecular studies confirm that site-specific gene expression profiles can predict treatment response patterns in approximately 73% of patients, providing a theoretical foundation for anatomically guided individualized treatment in the era of precision medicine (27).

CEA is a glycoprotein that is significantly elevated in patients with gastrointestinal tumors. It is currently the only internationally recognized tumor biomarker used to monitor the efficacy of CRC treatment (28). Further studies suggest that elevated CEA levels may be associated with inflammatory processes within the tumor microenvironment, which in turn create a conducive environment for tumor cell infiltration and metastasis. This finding is consistent with our results and is further supported by the SHAP model interpretation.

Research has shown that perineural invasion may be one of the key predictive indicators for lymph node metastasis in CRC. A study utilizing deep convolutional neural network technology to analyze pathological tissue images of CRC patients (29) assessed the predictive value of peritumoral stromal scores, including perineural invasion, for CRC lymph node metastasis. Additionally, perineural invasion is considered a critical driver of CRC progression (30). In clinical pathological feature analysis, observing whether the tumor exhibits perineural invasion is one of the independent factors for predicting lymph node metastasis and postoperative survival in CRC patients. Studies have found that the interaction between the tumor and nerves can jointly drive tumor development. From the perspective of tumor microenvironment construction, neurons release bioactive molecules such as neurotransmitters, angiogenesis-related factors, immune modulators, and growth factors, creating favorable conditions for tumor cell survival and proliferation. Moreover, neural networks provide potential biological pathways for tumor cell invasion and metastasis. Notably, tumor cells actively intervene in and regulate the tumor’s neural innervation by releasing nerve growth factors and guidance molecules. Further research has revealed that perineural invasion is an effective indicator for predicting the progression or recurrence of stage III CRC according to the Union for International Cancer Control (UICC) (31), and it also shows high predictive efficacy for local recurrence in rectal cancer. These findings underscore the critical role of perineural invasion in CRC prognostic evaluation.

It should be noted that the external validation cohort in this study included 185 stage III CRC patients from Shanxi Bethune Hospital, and there are differences in race, diet, and lifestyle compared to the SEER database. This population heterogeneity may affect the predictive accuracy of the model. Additionally, the sample size of the external validation cohort is relatively small, which may limit the generalizability of the validation results. We believe that the preliminary validation results in the Chinese patient cohort offer some reference value, but further prospective studies with larger and more diverse sample sizes are required to enhance the generalizability and reliability of the model, providing a more robust basis for the postoperative survival prognosis of stage III CRC patients in China.

In recent years, the application of machine learning models for CRC prognosis prediction has become increasingly widespread (9, 32), with the five-year survival status of stage III CRC patients after surgical treatment being a key clinical focus. This study emphasizes the interpretability of the model: machine learning algorithms can automatically identify key variables associated with cancer, allowing clinicians to intuitively understand the model’s decision-making process, thereby providing more precise diagnostic and treatment decision support (10). It provides scientific evidence for personalized predictions, enabling both clinicians and patients to understand potential risks. This study compared nine commonly used machine learning algorithms, including LR, DT, ENet, KNN, LightGBM, RF, XGBoost, SVM, and MLP. These algorithms are increasingly applied in tumor prognosis prediction, each with its own characteristics and suitable scenarios. Logistic regression offers good interpretability and stability, making it well-suited for datasets with clear linear relationships. However, its performance is limited when dealing with nonlinear associations or complex interactions among variables. Decision trees and random forests model data using a tree structure, which allows for handling nonlinearity and offers some robustness to missing data. However, they are prone to overfitting and generally lack interpretability (32). KNN relies on distance metrics between samples, making it sensitive to data normalization and computationally intensive in large datasets. SVM performs well with high-dimensional and sparse data, particularly in small sample settings, but is sensitive to parameter tuning and kernel selection. MLP, as a basic neural network structure, has strong nonlinear fitting capabilities but requires longer training time and lacks interpretability. In contrast, ensemble learning methods such as XGBoost and LightGBM demonstrate clear advantages in handling large-scale, nonlinear data, variable selection, and modeling speed. In particular, LightGBM employs histogram-based splitting and a leaf-wise growth strategy, which improves training efficiency while maintaining accuracy and generalizability (33). By comprehensively comparing prediction performance, interpretability, and clinical applicability, LightGBM outperformed the other models in this study. It achieved the highest AUC in the validation set and showed superior results across multiple performance metrics. Therefore, we selected LightGBM as the final prediction model and incorporated the SHAP method to enhance interpretability, providing a theoretical foundation for future clinical implementation. Previous studies also support the favorable performance of LightGBM in colorectal cancer prognosis prediction. Although the LightGBM model developed in this study demonstrated good overall predictive performance, misclassification analysis revealed limitations in its predictions for certain subgroups. False positives refer to patients who were actually alive but predicted by the model as deceased, which may lead to unnecessary psychological stress or overtreatment in clinical practice. Further analysis showed that these patients often exhibited high lymph node ratios (LNR) and T3/T4 stage tumors—factors typically associated with poor prognosis—but likely benefited from effective postoperative treatment, suggesting that the model may overestimate the lethality of certain risk factors. In contrast, false negatives—patients who were actually deceased but predicted as alive—pose more serious clinical consequences, such as insufficient monitoring or delayed intervention. We observed that some false negative patients had seemingly “low-risk” features, such as low LNR, negative CEA levels, or well-differentiated tumors, yet still experienced poor outcomes postoperatively. This indicates that the model has limitations in identifying atypical high-risk individuals. The misclassification analysis in this study highlights the model’s weaknesses in handling borderline cases and provides valuable guidance for future model refinement. It is recommended that future research incorporate additional variables that better reflect tumor biological behavior, or explore dynamic adjustment of decision thresholds to improve the model’s ability to identify critical misclassified cases, thereby enhancing its utility in personalized clinical decision-making.

Medical information technology is advancing rapidly, and the use of SEER data combined with machine learning methods to build predictive models offers a fresh perspective for CRC prognosis research. The scope of precision medicine and individualized treatment has been expanded, significantly improving the accuracy of clinical prediction models. While this study has achieved notable results, there are still some limitations: First, the data from the SEER database primarily originate from the United States, and racial and regional differences may result in findings that do not fully reflect the realities of patients in other countries or regions. The external validation cohort sample is relatively limited and may not be representative of patient characteristics across various regions in China. Second, the database does not provide key information on surgical treatment methods, postoperative complications, chemotherapy and radiotherapy regimens, or the sequence of treatments. Third, this is a retrospective study, and further prospective, multi-center, large-sample validation is required. Therefore, future studies should expand the clinical sample size and introduce more diverse data sources to enhance the model’s generalizability and predictive accuracy. Machine learning and artificial intelligence technologies continue to make breakthroughs. The integration of imaging features and biomarkers into multimodal frameworks will optimize CRC prognosis evaluation and provide reliable evidence for precision medicine. This technological pathway demonstrates significant potential in improving the efficiency of developing individualized treatment plans, particularly in addressing the challenges posed by tumor heterogeneity and therapeutic efficacy variations.

## 5 Conclusion

This study utilized SEER database resources to develop a machine learning framework, creating a prediction model for postoperative survival status in stage III CRC patients, with SHAP interpretation based on the LightGBM model. The model was validated using an independent external dataset, demonstrating stable predictive performance. Data analysis revealed that age, LNR, T stage, and chemotherapy intervention are key factors influencing postoperative survival prognosis, playing a significant role in cancer-specific survival outcomes. The LightGBM algorithm exhibited exceptional discrimination in time-to-event predictions, and its dynamic risk assessment functionality lays the foundation for the development of personalized follow-up plans. By developing a visual interactive interface, the model facilitates real-time analysis and visualization of prognosis risk, significantly enhancing the accuracy of clinical decision-making.

## Data Availability

The original contributions presented in the study are included in the article/**Supplementary Material**. Further inquiries can be directed to the corresponding authors.
