# Supplementary material for: Prediction of 5-year postoperative survival and analysis of key prognostic factors in stage III colorectal cancer patients using novel machine learning algorithms
Source: Front Oncol. 2025 Jul 14;15:1604386. doi: 10.3389/fonc.2025.1604386 (PMC12301204; doi:10.3389/fonc.2025.1604386)
Supplement: Supplementary file 1 [file Table1.docx]

Supplementary Table 1: Assignment of Values for Independent Variables

| Variable | Level |
| --- | --- |
| Marital status | Married = 1, Unmarried = 2, Unknown = 3 |
| Sex | Male = 1, Female = 2 |
| Tumor localization | Right colon = 1, Transverse colon = 2, Left colon = 3, Cecum-rectal junction = 4, Rectum = 5 |
| Pathological type | Adenocarcinoma = 1, Non-adenocarcinoma = 2 |
| T | T1 = 1, T2 = 2, T3 = 3, T4 = 4 |
| Radiation | Yes = 1, No = 2 |
| Chemotherapy | Yes = 1, No = 2 |
| Age | 14-65 years = 1, 66-80 years = 2, 81-89 years = 3 |
| Tumor size | 1-29mm = 1, 30-74mm = 2, 75-150mm = 3 |
| LNR | 0.01-0.11 = 1, 0.12-0.49 = 2, 0.50-1.00 = 3 |
| CEA | Positive = 1, Negative = 2 |
| PNI | Yes = 1, No = 2, Unknown = 3 |
| Grade | Grade Ⅰ = 1, Grade Ⅱ = 2, Grade Ⅲ = 3, Grade Ⅳ = 4, Unknown = 5 |
