# Supplementary figures and images for: Prediction of 5-year postoperative survival and analysis of key prognostic factors in stage III colorectal cancer patients using novel machine learning algorithms
Source: Front Oncol. 2025 Jul 14;15:1604386. doi: 10.3389/fonc.2025.1604386 (PMC12301204; doi:10.3389/fonc.2025.1604386)

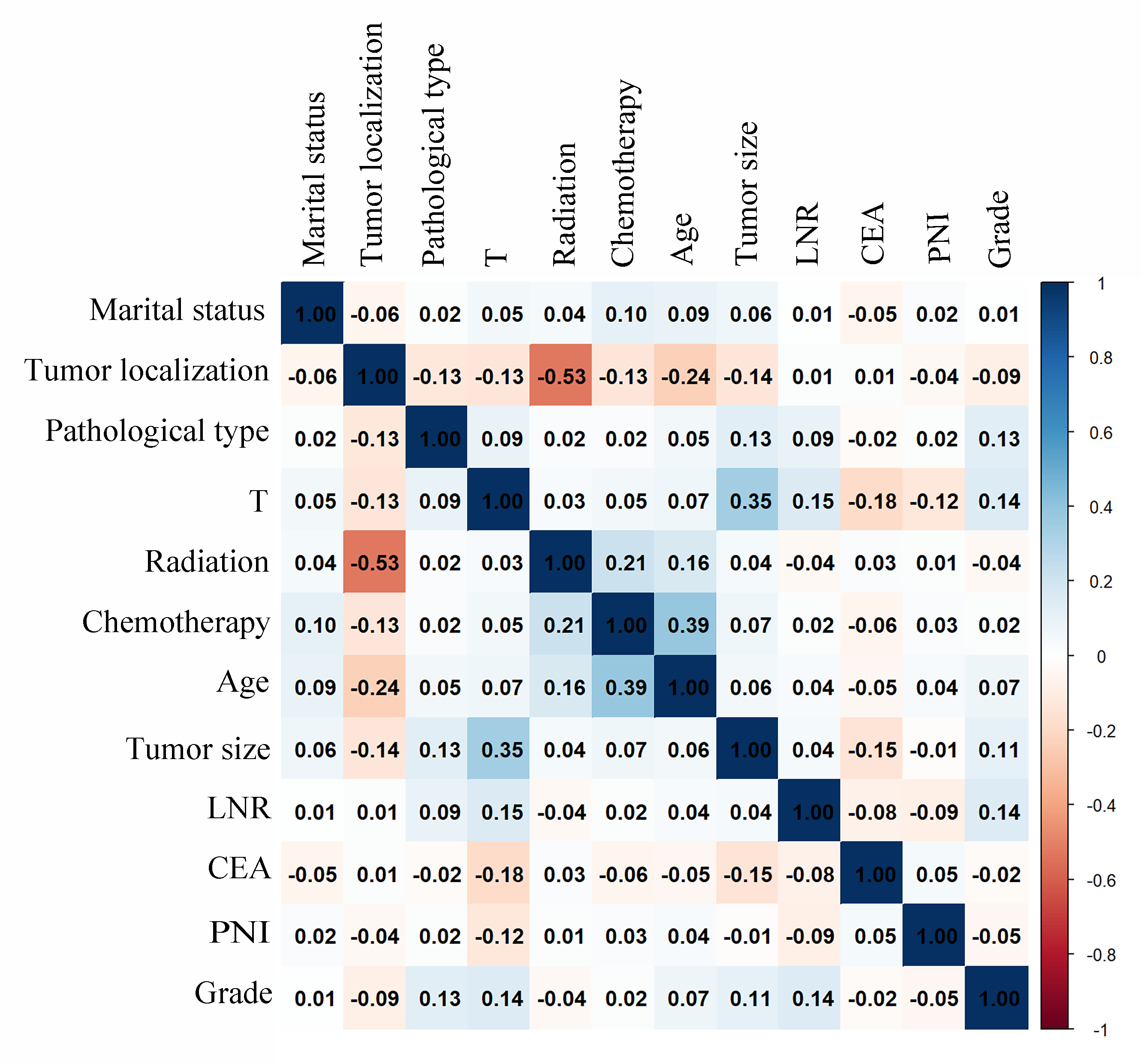

Supplement: Supplementary file 2 [file Image1.tif]

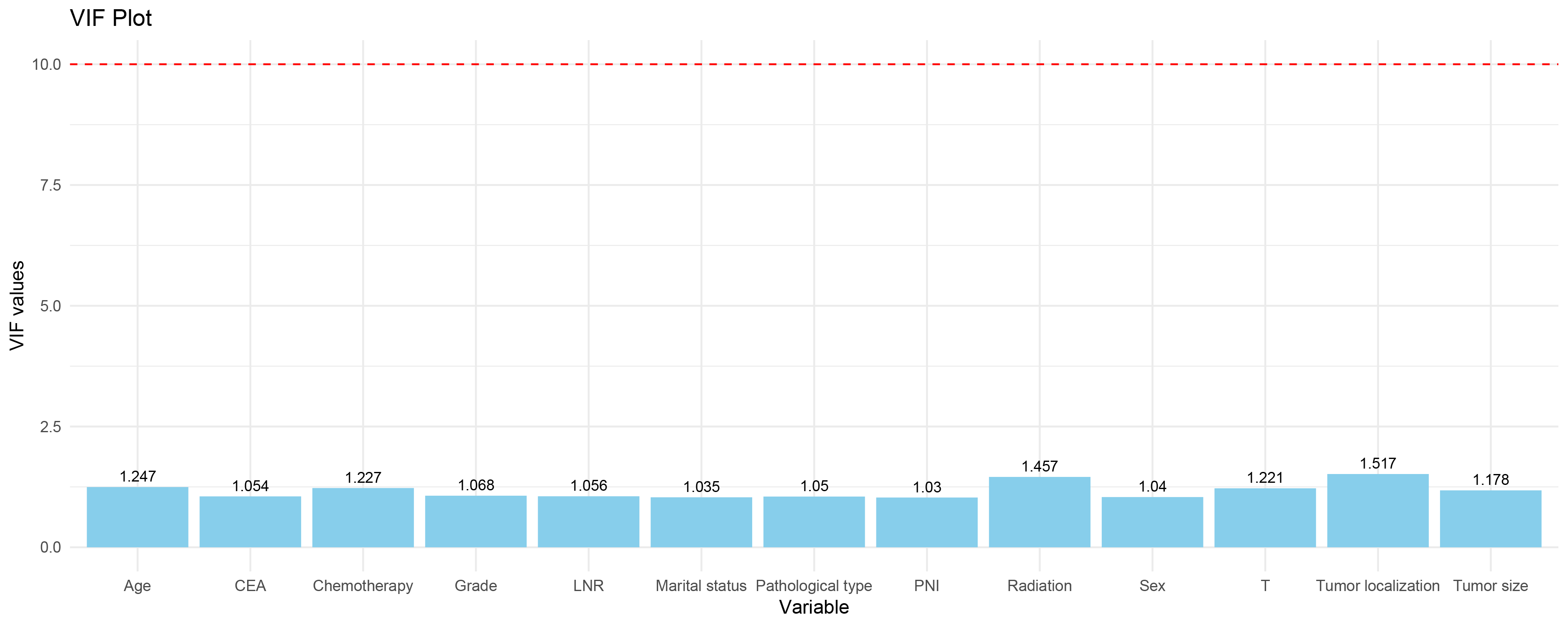

Supplement: Supplementary file 3 [file Image2.tif]
